# 879. Host Response Classifiers Identify Infection and Illness Severity and Improve Antibiotic Decision-Making in Patients with Suspected Infections Presenting to the Emergency Department

**DOI:** 10.1093/ofid/ofad500.924

**Published:** 2023-11-27

**Authors:** Arianna Beltran, Uan-I Chen, Edward A Michelson, Jay S Steingrub, Roger L Humphries, Jasreen K Gill, Alexandra Weissman, Evangelos J Giamarellos-Bourboulis, David W Wright, Oliver Liesenfeld, Natalie N Whitfield

**Affiliations:** University of Southern California School of Pharmacy, Los Angeles, California; Inflammatix, Sunnyvale, California; Texas Tech University Health Sciences Center, El Paso, Texas; Baystate Medical Center, Springfield, Massachusetts; University of Kentucky Chandler Medical Center, Lexington, Kentucky; Henry Ford Hospital, Detroit, Michigan; University of Pittsburgh/UPMC, Pittsburgh, Pennsylvania; National and Kapodistrian University of Athens, Medical School, Athens, Attiki, Greece; Emory University, Atlanta, Georgia; Inflammatix, Sunnyvale, California; Inflammatix, Sunnyvale, California

## Abstract

**Background:**

Nonspecific presentation and limited diagnostic solutions of acute infections and sepsis in emergency departments (EDs) result in early antimicrobial administration at a cost to antimicrobial stewardship. We validated the host response classifiers, IMX-BVN-3b and SEV-3b, to determine bacterial and viral infection status and illness severity. We also assessed antibiotic over- and underuse.

**Methods:**

We prospectively enrolled adult ED patients with suspected acute infections or sepsis with ≥1 vital sign abnormal across 7 sites. At enrollment, we surveyed treating physicians on infection probability and antibiotic prescribing. BVN/SEV-3b were calculated using NanoString nCounter® on PAXgene® blood samples. We compared BVN-3b likelihood of bacterial and viral infection to post-hoc clinical adjudication infection status and SEV-3b likelihood of severe outcomes to 7-day need for ICU care and 30-day mortality.

**Results:**

Of 568 enrolled patients, 346 had consensus adjudications (131 bacterial, 52 viral, 1 coinfection, 162 noninfected). The BVN-3b area under the receiver operating curve (AUROC) for bacterial infections was 0.82 (95%CI 0.77-0.87), compared to 0.76 (95%CI 0.71-0.81) for procalcitonin (p = 0.011). BVN-3b AUROC for viral infections was 0.89 (95%CI 0.84-0.95). SEV-3b AUROC was 0.78 (95%CI 0.69-0.86) and 0.88 (95%CI 0.73-1) for 7-day ICU care and 30-day mortality, respectively. Of the 346, 226 patients had a pre-lab result physician questionnaire; of these, 110 patients received antibiotics. Of these, BVN-3b would have corrected 71% of antibiotic prescribing errors (34/48 overprescriptions and 13/18 underprescriptions).

**Conclusion:**

BVN-3b and SEV-3b accurately identified bacterial and viral infections and risk status. If implemented in a rapid workflow, these tests may improve patient management and antibiotic prescribing, reducing healthcare costs in the ED.

**Disclosures:**

**Arianna Beltran, BS**, Inflammatix: Internship **Uan-I Chen, MS**, Inflammatix: Stocks/Bonds **Edward A. Michelson, MD**, Inflamatix: Research expense reimbursement for sponsored clinical trial **Alexandra Weissman, MD**, Inflammatix Inc: Advisor/Consultant **Evangelos J. Giamarellos-Bourboulis, PhD, D(ABMM)**, Abbot Product Operations AG: Grant/Research Support|Abbot Product Operations AG: Honoraria|bioMerieux: Grant/Research Support|bioMerieux: Honoraria|Horizon 2020 European Program: Grant/Research Support|Horizon Health European Program: Grant/Research Support|Sobi AB: Advisor/Consultant|Sobi AB: Grant/Research Support|Sobi AB: Honoraria **Oliver Liesenfeld, MD**, Inflammatix Inc.: Ownership Interest **Natalie N. Whitfield, PhD, D(ABMM)**, GenMark Dx/Roche: Employee|GenMark Dx/Roche: Stocks/Bonds|Inflammatix: Employee|Inflammatix: Stocks/Bonds

